# Epitaxy‐Directed Self‐Assembly of Copolymers and Polymer Blends

**DOI:** 10.1002/advs.202207707

**Published:** 2023-11-23

**Authors:** Chunyue Hou, Junjie Wang, Peng Wang, Jiahui Cui, Shaojuan Wang, Rui Xin, Huihui Li, Xiaoli Sun, Zhongjie Ren, Shouke Yan

**Affiliations:** ^1^ Key Laboratory of Rubber‐Plastics Qingdao University of Science and Technology Qingdao 266042 China; ^2^ State Key Laboratory of Chemical Resource Engineering Beijing University of Chemical Technology Beijing 100029 China

**Keywords:** copolymers, epitaxy, patterned structure, polymer blends, self‐assembly

## Abstract

Directed self‐assembly of materials into patterned structures is of great importance since the performance of them depends remarkably on their multiscale hierarchical structures. Therefore, purposeful structural regulation at different length scales through crystallization engineering provides an opportunity to modify the properties of polymeric materials. Here, an epitaxy‐directed self‐assembly strategy for regulating the pattern structures including phase structure as well as crystal modification and orientation of each component for both copolymers and polymer blends is reported. Owing to the specific crystallography registration between the depositing crystalline polymers and the underlying crystalline substrate, not only order phase structure with controlled size at nanometer scale but also the crystal structure and chain orientation of each component within the separated phases for both copolymers and polymer blend systems can be precisely regulated.

## Graphical abstract

The epitaxy‐directed autonomous self‐assembly of alternatively arranged and alternatively distributed PCL and PEG edge‐on lamellar structures with temperature‐controlled domain size in ultrathin films of PCL‐*b*‐PEG diblock copolymers on highly oriented PE and iPP substrates, respectively, is reported. The arrows show the molecular chain directions of melt‐drawn oriented PE and iPP substrate films.

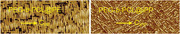

## 1 Introduction

Directed self‐assembly of materials into patterned structures is very important for many diverse areas of nanotechnology, such as, optoelectronics and sensing devices.^[^ ^1^, ^2^, ^3^, ^4^ ^]^ This has stimulated mass researches on the self‐assembly of block copolymers (BCPs). It is now well known that BCPs can be organized into a variety of morphologies depending on volume fraction and sequence length of the blocks, the compatibility between the components, film thickness, nature of the substrate, and preparation condition, etc.^[^ ^5^, ^6^, ^7^, ^8^ ^]^ A key challenge in this field is to create well‐controlled unique structures of the self‐assembled entities. Therefore, various methods have been developed to control the uniformity and directionality through molecular interactions and/or external fields.^[^ ^4^, ^9^, ^10^, ^11^, ^12^, ^13^ ^]^ Among them, the combination of bottom‐up self‐assembly with “top‐down” patterned templates, that is, directed self‐assembly (DSA), is confirmed to offer great opportunity for a source of innovation in nanofabrication.^[^ ^14^ ^]^ The orientation and placement of BCP domains are directed by topographically or chemically patterned templates. The specific organization of the molecules is realized though either graphoepitaxy or colloidal particles depending on the commensurability between the sphere diameter and length scale of the template.

An advantage of the DSA method is the arbitrary geometrical design and the superior nanometer‐level precision of the templates. The lack of specific crystallography registration of deposited polymers to the templates makes, however, difficult to purposefully control the crystal modification of polymorphic polymers. In contrast to DSA method, epitaxy based on crystallographic matching of depositing crystalline polymers with the underlying crystalline substrates exhibits specific crystallography registration between every small molecule or chain segment of polymers in crystalline state and the substrate.^[^ ^15^, ^16^ ^]^ It can, therefore, control the orientation and position of atoms, molecules, or chain segments of polymers in the depositing layer precisely through crystallographic interactions. Taking this into account, the epitaxy‐directed crystal growth possesses the following advantages in the structure control of polymeric materials. i) The substrate can be any kind of crystalline materials, such as inorganic and organic single crystals as well as highly oriented polymer thin films,^[^ ^17^, ^18^, ^19^ ^]^ which makes an easy fabrication and diversity selection of the templates. The substrate and the overgrowth layer can even crystallize synchronously during a single process as long as the substrate crystals form prior to the nucleation of the over layer.^[^ ^20^ ^]^ ii) It can lead to a polymorphic polymer crystallizes in a desired crystal modification but with different molecular chain and crystal orientation.^[^ ^21^, ^22^, ^23^, ^24^, ^25^ ^]^ Occasionally, a combination of heteroepitaxy induced by used foreign substrate and homoepitaxy triggered by early formed epitaxial crystals can even create more complicated pattern structures.^[^ ^26^ ^]^ iii) The epitaxy‐directed crystal growth can be achieved from different initial states including vapor, amorphous glassy, and melt phases as well as by solution crystallization,^[^ ^21^, ^22^, ^23^, ^24^, ^25^, ^26^, ^27^ ^]^ which endows the self‐repairing capability of the oriented structures simply through melt‐recrystallization.^[^ ^28^ ^]^ This benefits for different processes and is of great significance to extend their lifetime and reduce maintenance costs. iv) The epitaxy‐directed crystal growth can even be realized during in situ polymerization,^[^ ^29^ ^]^ which can simplify the fabrication process and avoid the processing‐caused structural damage or foreign impurities.

In spite of the aforementioned advantages, there is relatively little research on epitaxy directed crystallization of copolymers. De Rosa et al.^[^ ^30^, ^31^ ^]^ have first studied the self‐organization of BCPs containing crystallizable polyethylene (PE) blocks. Based on the directional solidification from eutectic solution of crystallizable organic solvent for the strongly segregated semicrystalline BCPs, large area 2D periodic thin films with uniformly aligned cylindrical domains have been fabricated. They have further conducted the epitaxy‐template‐crystallization of a crystalline–crystalline BCP containing PE and syndiotactic polypropylene (sPP) blocks, that is, PE‐*b*‐sPP copolymer on *p*‐terphenyl single crystal surface.^[^ ^32^ ^]^ It has been demonstrated that oriented lamellar structure of both PE and sPP blocks can be produced. The orientation of both blocks depends on the crystallization sequence. When sPP crystallized first, parallel‐aligned lamellar structure of both sPP and PE was observed. On the other hand, cross‐hatched lamellar structure was obtained if PE block crystallizes before sPP. This is related to the intrinsic different epitaxial orientation of sPP and PE on the *p*‐terphenyl crystals in the way that the epitaxially crystallized block determines the orientation of the other block via confined crystallization of it. Here, we demonstrate the capability of epitaxy to direct the alignment of not only crystalline–crystalline BCPs but also polymer blends. Considering the wide applications of polymer blends, such as the donor and acceptor in photovoltaic devices, the self‐assembly of polymer blends into patterned structures is of even great importance. To this end, poly(ε‐carolactone)‐*b*‐methoxy‐poly(ethylene glycol) (PCL‐*b*‐PEG) diblock and poly(ε‐carolactone)‐*b*‐poly(butylene adipate)‐*b*‐poly(ε‐carolactone) (PCL‐*b*‐PBA‐*b*‐PCL) triblock copolymers with different block lengths were synthesized. For direct comparison, the capability of oriented alignment of PCL/PBA blend through epitaxial crystallization was also checked.

## 2 Results and Discussion

### 2.1 Epitaxy‐Directed Self‐Assembly of PCL‐*b*‐PBA‐*b*‐PCL on Oriented PE Films

#### 2.1.1 Morphologies of PCL and PBA Grown on Oriented PE Thin Films

**Figure 1:**
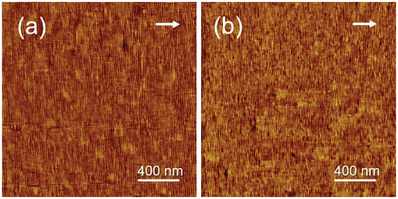
AFM phase images of PCL (a) and PBA (b) homopolymers crystallized isothermally on oriented PE thin films at 30 °C for 24 h after melting at 80 °C for 10 min. The white arrows indicate the molecular chain direction of oriented PE films.

The epitaxial crystallization of PCL and PBA homopolymers on oriented PE has been reported previously.^[^ ^21^, ^33^, ^34^ ^]^ For the completeness and legibility of this article, **Figure** **1** presents the representative AFM phase images showing the morphologies of PCL (Figure 1a) and PBA (Figure 1b) crystallized on oriented PE thin films. It is clear that the oriented PE substrate can induce epitaxial crystallization of both PCL and PBA, which results in a parallel alignment of PCL and PBA edge‐on lamellae perpendicular to the PE molecular chain direction, i.e., a parallel chain arrangement of both PCL and PBA along the PE chain direction, which has been supported by the corresponding electron diffraction via the same orientation of (00l) reflections for PCL, PBA, and PE.^[^ ^21^, ^33^ ^]^ Therefore, highly oriented edge‐on lamellar structure is expected also for the PCL and PBA blocks in the PCL‐*b*‐PBA‐*b*‐PCL triblock copolymers.

#### 2.1.2 Morphologies of PCL‐*b*‐PBA‐*b*‐PCL Crystallized on Oriented PE Thin Films

**Figure 2:**
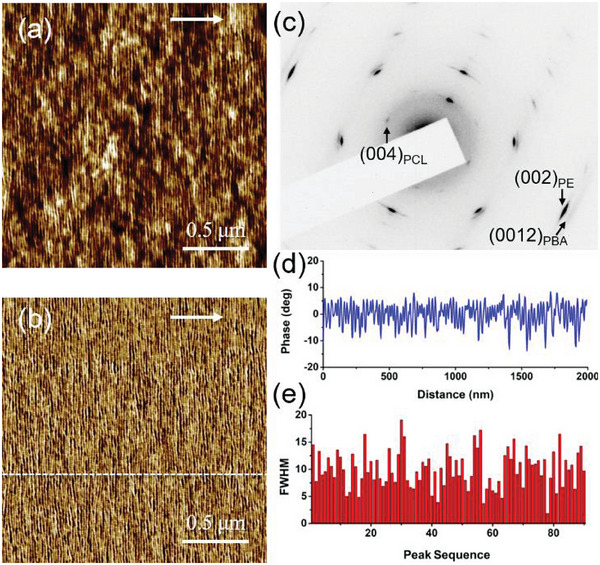
a,b) AFM height and phase images of the PCL(13.5k)‐*b*‐PBA(11k)‐*b*‐PCL(13.5k) triblock copolymer grown epitaxially on highly oriented PE substrate at 37.5 °C after melting at 80 °C for 10 min. The white arrows in the AFM images indicate the molecular chain direction of the PE substrate. c) A corresponding electron diffraction pattern of the sample. d) The height profile corresponding to the white line shown in part (b). e) The FWHM distribution obtained from part (d).

**Figure** **2** shows the AFM height and phase images of a PCL(13.5k)‐*b*‐PBA(11k)‐*b*‐PCL(13.5k) triblock copolymer grown isothermally on highly oriented PE substrate at 37.5 °C, respectively. Parallel aligned edge‐on lamellar structure can be clearly seen in both the height (Figure 2a) and phase (Figure 2b) images, indicating the occurrence of epitaxial crystallization of both PCL and PBA blocks on PE substrate, resulting in the directed self‐assembly of the copolymer. The corresponding electron diffraction pattern shown in Figure 2c displays the well‐defined reflection spots of both PCL and PBA blocks as well as the highly oriented PE substrate, confirming the high orientation of both PCL and PBA blocks in the copolymer on the oriented PE film. Moreover, the alignment of (00l) diffraction spots for PCL and PBA crystals in the same direction of PE (002) diffraction demonstrates the same crystallographic *c*‐axis orientation of PCL and PBA crystals with PE ones, i.e., the parallel chain alignment of PCL and PBA blocks in the copolymer along the PE chain like each homopolymer. From the AFM height profile shown in Figure 2d, it can be seen that the parallel aligned PCL and PBA lamellae exhibit nice periodicity in nanometer scale. The corresponding full width at half‐maximum (FWHM) distribution obtained from Figure 2d is presented in Figure 2e.

It should be noted that the PE substrate caused self‐assembly of PCL‐*b*‐PBA‐*b*‐PCL BCPs is not block length dependent, as shown in Figure S1 (Supporting Information). Also the polymorphic behavior PBA blocks in the copolymers has been controlled, i.e., the growth of β‐PBA crystals regardless of crystallization temperature, like the PBA homopolymer grown epitaxially on oriented PE substrate.^[^ ^21^ ^]^ Moreover, the patterned thin film exhibits a quite smooth surface with an ultralow surface roughness of only ≈1.4 nm (see **Table** **1**), which is reported to be crucial for many systems, such as Low Voltage Non‐Volatile Polymer Memory.^[^ ^35^ ^]^ Most importantly, the long period and surface roughness of the unique oriented structure can be simply regulated by controlling the crystallization temperature. As summarized in Table 1, with decreasing crystallization temperature, the long period decreases from 22 nm (grown at 37.5 °C) to 19.8 nm (grown at 32.5 °C), while the roughness declines from 1.43 to 1.30 nm. The decrease of long period with decreasing crystallization temperature rests on the dependence of lamellar thickness on crystallization temperature according to the L−H model,^[^ ^36^ ^]^ i.e., Equation (1):

(1) $$l_{c}=2\sigma_{e}T_{m}^{0}/\left(T_{m}^{0}-T_{c}\right)\Delta H_{m}+c$$

where $T_{m}^{0}$ is the equilibrium melting temperature, σ_e_ is the folding surface free energy, Δ*H*_m_ is the heat of fusion, *c* is a constant, and *l*_c_ is the lamellar thickness obtained at crystallization temperature *T*_c_.

**Table 1:** Temperature‐dependent long period and roughness of PCL–PBA triblock copolymer grown on oriented PE substrate.

Temperature [°C]	Long period [nm]	Roughness [nm]
37.5	22.0^a^	1.43^a^
32.5	19.8^b^	1.30^b^

^a)^ Obtained according to Figure 2

^b)^ Obtained according to Figure S2 (Supporting Information).

### 2.2 Directed Self‐Assembly of PCL‐*b*‐PEG Copolymer on Oriented PE Film

**Figure 3:**
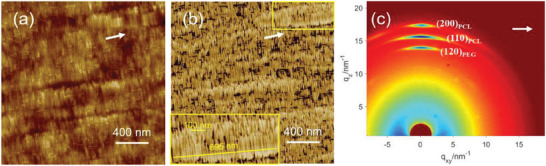
a,b) AFM height and phase images of a PCL(50k)‐*b*‐PEG(1.9k) diblock copolymer grown epitaxially on highly oriented PE substrate at 40 °C after melting at 80 °C for 10 min. c) A corresponding WAXD pattern of the sample after removing the PE substrate taken at room temperature. The white arrows indicate the molecular chain direction of the PE substrate.

The self‐assembly of PCL‐*b*‐PBA‐*b*‐PCL copolymer on oriented PE film is based on the epitaxial capability of both PCL and PBA blocks on highly oriented PE substrate with exactly the same crystallographic orientation feature. One may argue that both blocks of a copolymer exhibiting exactly the same mutual chain orientation relationship with one substrate is seldom satisfied, and thus the method can be applied only to very limited systems. This is actually not the case. We have confirmed that it is also effective for copolymers with only one block can grow epitaxially. Here, the PCL‐*b*‐PEG diblock copolymer has been taken as an example. It is confirmed that the PE substrate cannot induce the epitaxial crystallization of PEG. As presented in Figure S3 (Supporting Information), the PEG grown on PE substrate shows the same spherulitic morphology as on glass slide surface, indicating the incapability of PE for inducing PEG epitaxial crystallization. However, as presented in **Figure** **3**a,b, the PCL(50k)‐*b*‐PEG(1.9k) diblock copolymer crystallized on oriented PE substrate creates also a parallel aligned edge‐on lamellar structure similar to that of the PCL–PBA triblock copolymer. The corresponding wide angle X‐ray diffraction (WAXD) pattern shown in Figure 3c confirms the orientation of both blocks in copolymer unambiguously. In the WAXD pattern, the inner most diffraction arc is contributed by (120) lattice planes of PEG block, which disappears at 54 °C (Figure S4, Supporting Information) since it has a lower molecular weight of only 1.9k and thus a relatively lower melting point. The appearance of (120)_PEG_, (110)_PCL_, and (200)_PCL_ in the same direction perpendicular to the PE chain direction demonstrates a parallel chain alignment of both PCL and PEG blocks along PE chain direction. The inset of Figure 3b shows an enlarged part marked by a yellow rectangle in upper‐right corner. It is measured that the length corresponding to 43 parallel aligned lamellae is ≈695 nm. This means that the periodicity of the parallel arranged lamellae is ≈16.2 nm.

**Figure 4:**
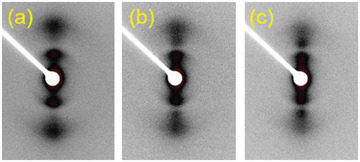
2D SAXS patterns of PCL(50k)‐*b*‐PEG(1.9k) diblock copolymer grown epitaxially on highly oriented PE substrate at 40 °C taken at room temperature (a), and during heating at 50 °C (b), and 55 °C (c), respectively.

To figure out the specific distribution of PCL and PEG blocks in the film grown on the PE substrate, small angle X‐ray scattering (SAXS) experiments were conducted. **Figure** **4** presents the 2D SAXS patterns of the PCL(50k)‐b‐PEG(1.9k) diblock copolymer grown on PE but after removing the PE substrate taken at different temperatures. The appearance of second‐order scattering in the SAXS pattern indicates a high degree of crystal orientation for both PCL and PEG blocks. To get quantitative information about the periodic structure, the 2D‐SAXS patterns shown in Figure 4 have been converted into 1D‐SAXS profiles as presented in Figure S5 (Supporting Information). According to the second peak of the 1D‐SAXS profile taken at room temperature shown in Figure S5a (Supporting Information) a long period of 16.5 nm has been obtained. It corresponds clearly to the periodicity of the parallel arranged alternative PCL and PEG lamellae shown in Figure 3b. The second peak disappears at 55 °C due to the melting of PEG crystals while the first peak remains essentially unchanged. The long period obtained from the first peak is approximately 31 nm, which is about two times of that acquired from the second peak. This refers unambiguously an alternatively arranged PCL and PEG lamellar structure. This illustrates the capability of epitaxy‐directed self‐assembly for fabricating perpendicularly and alternatively arranged lamellar phase separation structure of copolymers with even only one component exhibiting epitaxial crystallization ability. It should be noticed that the oriented self‐assembly of PCL‐*b*‐PEG diblock copolymers on PE substrate is also independent of PCL and PEG sequence length (Figure S6, Supporting Information) and the phase size (i.e., lamellar thickness or long period) is directly related to the crystallization temperature as well.

### 2.3 Directed Self‐Assembly of PCL/PBA Blend on Oriented PE Film

**Figure 5:**
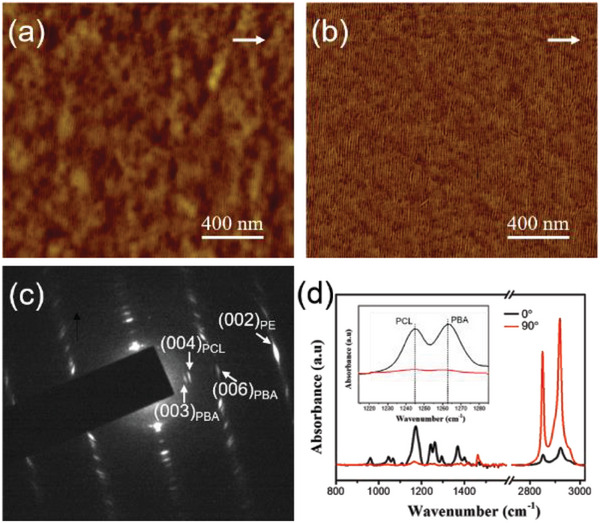
a,b) AFM height and phase images of a PCL/PBA (50/50 wt.%) blend film crystallized isothermally on oriented PE substrate at 20 °C. The white arrows indicate the molecular chain direction of PE. c) A corresponding electron diffraction pattern and d) polarized FTIR spectra measured with the electron vectors parallel (0°) and perpendicular (90°) to the PE chain direction, respectively.

It should be mentioned that the synergistic control of phase separation behavior and intrinsic structures of each component for blend systems is also very important in many cases. For example, Shah and Ganesan reported that the most desirable morphology for a photovoltaic device based on semiconductive polymers is the perpendicularly oriented and alternatively distributed donor and acceptor lamellar phases with optimal domain size of 10–25 nm.^[^ ^37^ ^]^ This has been confirmed to be successfully realized by epitaxy‐directed self‐assembly. Taken the PCL/PBA blend on oriented PE substrate as an example, **Figure** **5**a,b shows the AFM height and phase images of a PBA/PCL (50/50 wt.%) blend crystallized isothermally on oriented PE substrate at 20 °C. Periodically aligned edge‐on lamellar structure of PBA/PCL blend on PE substrate can be clearly observed with lamellae oriented perpendicular to the molecular chain direction of PE. The corresponding electron diffraction pattern (Figure 5c) shows the well‐defined reflection spots of PBA, PCL, and PE, demonstrating the high degree of orientation of both PBA and PCL components in the blend on ordered PE substrate. The appearance of (003)_PBA_, (004)_PCL_, and (002)_PE_ diffractions in the same direction confirms a parallel alignment of both PBA and PCL molecular chains along the PE chain direction. This is further confirmed by the polarized infrared spectra shown in Figure 5d. Furthermore, according to Figure 5d, an orientation function can be obtained by Equation (2).^[^ ^23^, ^38^ ^]^

(2) $$f\;=\frac{I_{//}/I_{⊥}-1}{I_{//}/I_{⊥}+2}\;\times \frac{2}{3\cos^{2}a-1}\;$$

where *I*_//_ and *I*_⊥_ are the intensities of the interested band measured with electron vectors parallel (0°) and perpendicular (90°) to the reference direction (here the molecular chain direction of PE substrate), while *α* is the angle between the transition moment vector of the used band and the molecular chain axis, which is 90° for both the 1245 cm^−1^ band of PCL and 1263 cm^−1^ band of PBA. The obtained orientation functions of PCL and PBA are 0.87 and 0.86, respectively, reflecting a high degree of orientation for both of PCL and PBA.

**Figure 6:**
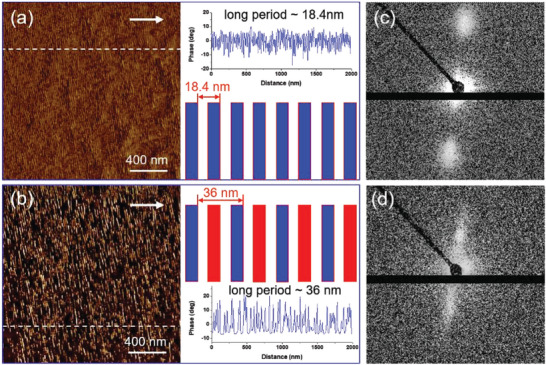
AFM phase images of a PCL/PBA (50/50 wt.%) blend crystallized on the oriented PE substrate isothermally at 25 °C recorded at room temperature (a) and at 54 °C after selective melting of PBA (b). The white arrows indicate the PE molecular chain. The corresponding phase profles along the white lines in the phase images and sketches illustrating the structures with long periods of 18.4 and 36 nm before and after melting of PBA crystals (right panels of parts (a) and (b)). The molten PBA crystals are indicated by the red rectangles. 2D SAXS patterns taken at room temperature (c) and at 54 °C (d), respectively.

The above AFM and polarized FTIR results demonstrate the epitaxial crystallization of both components in PCL/PBA blend on oriented PE substrate, which result in the ordered edge‐on lamellar structure. They can, however, not provide the information about the phase distribution of PCL and PBA. To find out this, small angle X‐ray scattering (SAXS) and AFM experiments during selective melting of PBA were conducted. As presented in **Figure** **6**, the AFM phase image taken at room temperature (Figure 6a) shows tightly stacked edge‐on lamellae. The corresponding phase profiles along the white lines in the phase image demonstrate a long period of the sample crystallized at 25 °C to be ≈18.4 nm, which becomes ≈36 nm after selective melting of the PBA crystals at 54 °C (Figure 6b. An approximately doubled long period after melting of PBA indicates an alternative distribution of the PCL and PBA lamellae as schematically displayed in the right panel below the phase profiles, i.e., the formation of an alternatively arranged PCL and PBA lamellar structure. This has further been confirmed by the SAXS conducted at room temperature and 54 °C (Figure 6c,d). Even though the calculated long periods from SAXS of 18.7 and 36.5 nm, respectively, are slightly larger than those obtained from AFM results, the multiplied long period after melting of PBA supports the alternative arrangement of the PCL and PBA lamellae.

The perpendicular and alternative arrangement of the PCL and PBA lamellae has the following advantages. First, as summarized in **Table** **2**, the long period of the lamellar pattern, i.e., the domain size of PCL and PBA, can be simply regulated by control the crystallization temperature. This rests on the crystallization‐temperature‐dependent PCL and PBA lamellar thicknesses, namely the higher the temperature, the thicker the lamellar thickness. Second, it is well documented that the PBA is a polymorphic polymer exhibiting α and β phases grown at high (>40 °C) and low (<28 °C) temperatures during melt crystallization, respectively. By crystallizing the PBA/PCL blend on oriented PE substrate, except for the same parallel chain alignment of PCL and PBA along PE chain direction, the PBA crystallizes always in its β phase regardless of temperature.

**Table 2:** Temperature‐dependent long period of PCL/PBA blend grown on PE.

Temperature [°C]	Long period [nm]
20^a^ ^)^	14.3
25^b^ ^)^	18.7
30^c^ ^)^	20.5
40^d^ ^)^	23.8

^a)^ See Figure 4

^b)^ See Figure 5

^c)^ See Figure S7a (Supporting Information)

^d)^ See Figure S7b (Supporting Information).

### 2.4 Directed Self‐Assembly of PCL‐*b*‐PEG Copolymer on Oriented iPP

**Figure 7:**
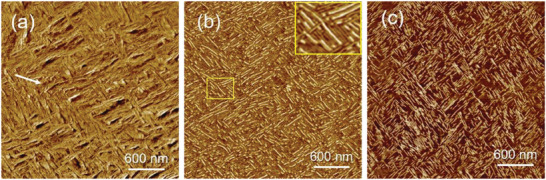
AFM images of PCL(10k)‐*b*‐PEG(10k) scanned at a) room temperature, b) 56 °C, c) and 58 °C, respectively. The inset in (b) is an enlarged image of the part marked by the yellow rectangle. The white arrow indicates the molecular chain direction of the used iPP substrate.

All of the examples shown above illustrate the ability of epitaxy‐directed self‐assembly of copolymers and polymer blends for generating parallel‐aligned lamellar structure with tunable domain size of alternatively and perpendicularly separated phases, as well as the crystal modification and parallel chain alignment of each component. The epitaxy‐directed self‐assembly has also another advantage. As emphasized in the introduction part, it can realize different molecular chain and crystal orientation of deposit polymer depending on the specific crystallographic interaction of it with the substrate crystals.^[^ ^21^, ^22^, ^23^, ^24^, ^25^ ^]^ We have taken the PCL‐*b*‐PEG copolymer as an example again and checked the crystalline morphology of it grown on an oriented iPP substrate. It is well documented that epitaxial crystallization of PCL on oriented iPP substrate results in the formation of a cross‐hatched lamellar pattern with the PCL lamellae ± 40° apart from the molecular chain direction of iPP as illustrated in Figure S8 (Supporting Information).^[^ ^34^, ^39^ ^]^ In other words, the PCL and iPP chains are inclined to an angle of ± 50°. On the other hand, it has been confirmed that the PEG does not exhibit epitaxial ability on iPP substrate as well. **Figure** **7** presents AFM phase images of a PCL(10k)‐*b*‐PEG(10k) copolymer crystallized from its 0.5 wt.% chloroform solution spin‐coated on the surface of an oriented iPP substrate. Like the PCL grown on the iPP substrate (Figure S8, Supporting Information), similar cross‐hatched lamellar structure of PCL‐*b*‐PEG copolymer is observed on the oriented iPP substrate (Figure 7a). To reveal the exact phase structure of the copolymer, the obvious lamellar thickening of PEG, which not evident for PCL (cf. Figure S9, Supporting Information), during heating was utilized. As presented in Figure 7b, when heating the sample shown in Figure 7a from room temperature up to 56 °C, the AFM phase image becomes more clearly due to the structure reorganization or secondary crystallization. Moreover, owing to the lamellar thickening of PEG block, the phase structure can now be clearly identified (see the enlarged inset of Figure 7b). It is evident that the epitaxial crystallization of PCL on iPP substrate promotes a similar organization of the PEG blocks even though without epitaxy ability on iPP, which is more clearly seen after the melting of crystals corresponding to PCL blocks at 58 °C (Figure 7c). It should be emphasized that similar cross‐hatched lamellar assembly can be reconstructed after melting of both components and recrystallization during cooling, indicating its independence of crystallization pathway.

## 3 Conclusion

In summary, crystallization of PCL‐*b*‐PEG diblock and PCL‐*b*‐PBA‐*b*‐PCL triblock copolymers as well as PCL/PBA blends on highly oriented PE and/or iPP substrates has been studied. It was found that the crystallization of PCL‐*b*‐PBA‐*b*‐PCL triblock copolymers and PCL/PBA blends on highly oriented PE substrate results in the self‐assembly of both components into patterned structures with parallel oriented lamellae owing to the capability of epitaxy for both PCL and PBA with PE. The oriented lamellae of PCL and PBA are confirmed to be alternatively arranged with molecular chains or chain segments aligned in the same direction of oriented PE chains, which is ideal for the donor and acceptor phases of photovoltaic device based on semiconductive polymers. Most importantly, the separated domain size of PCL and PBA can be easily regulated by controlled crystallization temperature. Even though both oriented PE and iPP substrates lack the epitaxial capacity toward the PEG block in the PCL‐*b*‐PEG diblock copolymers, similar perpendicularly separated and alternatively distributed PCL and PEG lamellae with temperature‐dependent thicknesses aligned normal to the PE chain direction are produced when crystallizing on oriented PE substrate. Moreover, the change of substrate from PE to iPP leads to the molecular chains of both PCL and PEG in the perpendicularly separated lamellar domains ± 50° apart from the chain direction of iPP substrate. All these manifest the possibility of epitaxy‐directed self‐assembly strategy for regulating the patterned structures including both phase structure with controlled size at nanometer scale as well as crystal modification and orientation of each component of copolymers and polymer blends.

## 4 Experimental Section

### Materials

High‐density PE was obtained from Lanzhou Petrochemical, China. IPP was produced by Yanshan Petroleum and Chemical Company, China. PBA (weight‐average molecular weight of ≈4 × 10^4^ g mol^−1^ and polydispersity of 1.7) and PCL (weight‐average molecular weight of ≈6.5 × 10^4^ g mol^−1^ and polydispersity of 1.5) used for studying the epitaxy of homopolymers on oriented substrate were produced by BASF AG Ludwigshafen, Germany. The xylene, toluene, and chloroform solvents were purchased from Beijing Chemical Reagent Co., Ltd. and used without further purification.

### Synthesis of PCL‐b‐PEG Diblock Copolymer

To obtain the block copolymers of PEG‐*b*‐PCL, the stannous octoate was adopted as catalyst (0.1 mol% of the amount of ε‐caprolactone) and methoxypolyethylene glycols with different molecular weights, for example, 1900, 10 000, and 20 000, were used to serve as initiators to achieve the ring opening polymerization of ε‐caprolactone. The length of PCL segment has been regulated by controlling the feed ratio of ε‐caprolactone monomer. The mixture of reagents was dissolved into anhydrous toluene (20 mL) under Ar atmosphere and stirred at 130 °C for 24 h. After that, the reaction system was cooled to room temperature, and extra toluene solvent was removed by vacuum rotatory evaporator. The resultant crude product was then re‐dissolved by chloroform solvent, and subsequently dropped into cold diethyl ether/*n*‐hexane (1/1) to gain precipitation. After suction filtration and re‐dissolution for three times, the final precipitation was further purified by Soxhlet using *n*‐hexane as extractor and thoroughly dried in vacuum oven at 40 °C for 24 h to gain the product of PEG‐*b*‐PCL. Five kinds of block polymers, i.e., PEG(1.9k)–PCL(5k); PEG(1.9k)–PCL(10k), PEG(1.9k)–PCL(20k), PEG(10k)–PCL(10k), and PEG(20k)–PCL(20k), were obtained by changing feed ratio. The corresponding ^1^H NMR spectra (400 MHz, CDCl_3_) *δ* 4.08 (br), 3.66 (s), 3.40 (s), 2.33 (br), 1.67 (br), 1.41 (br) are presented in Figure S10 (Supporting Information).

### Synthesis of Poly(butylene adipate) Diol (PBA)

To prepare PCL‐*b*‐PBA‐*b*‐PCL triblock copolymers, hydroxyl capped PBA block was first synthesized in the following way. A suitable amount of mixture of adipic acid and 1, 4‐butanediol at a molar ratio of 1:1.05 was added into reaction flask, followed by dropping tetrabutyl titanate (0.02 mL) catalyst into the reaction system under stirring. The manifold instrument and condenser pipe were connected with reaction flask in sequence. After stirring at 180 °C for 12 h under Ar atmosphere, the manifold instrument and condenser pipe were removed, and the mixture was further stirred at 180 °C for another 4 h under low pressure condition. When the reaction systems were cooled down to room temperature, a suitable amount of chloroform was added into reaction system to dissolve the product, and then dropped into cold *n*‐hexane to gain precipitation. After suction filtration and re‐dissolution for three times, the final precipitation was further purified by Soxhlet using *n*‐hexane as extractor and thoroughly dried in vacuum oven for 24 h to gain the PBA product for further use. The corresponding ^1^H NMR spectrum (400 MHz, CDCl_3_) *δ* 1.62‐1.73 (br), 2.30‐2.35 (br), 4.06 (br), 6.8–8.0 (─OH, br) is shown in Figure S11 (Supporting Information). The number average molecular weight determined by end group to be *M*_n_ = 10 956.

### Synthesis of PCL‐b‐PBA‐b‐PCL Triblock Copolymers

The synthesis of PCL‐*b*‐PBA‐*b*‐PCL block copolymers was performed in a similar process as that of PEG‐*b*‐PCL, except for using the hydroxyl capped PBA as initiator for ring opening polymerization of ε‐caprolactone. The stannous octoate (0.02 mL) was also adopted as catalyst. Four copolymers, i.e., PCL(54.5k)–PBA(11k)–PCL(54.5k), PCL(22k)–PBA(11k)–PCL(22k), PCL(13.5k)–PBA(11k)–PCL(13.5k), and PCL(3.5k)–PBA(11k)–PCL(3.5k), were obtained by changing feed ratio. The related ^1^H NMR spectra (400 MHz, CDCl_3_) *δ* 1.33–1.43(br), 1.60–1.73(br), 2.25–2.45(br), 4.06–4.20(br) can be found in Figure S12 (Supporting Information).

### Sample Preparation

The highly oriented PE and iPP ultrathin films were prepared by a melt‐draw technique introduced by Petermann and Gohil.^[^ ^40^ ^]^ According to this method, a small amount of 0.5 wt.% solution in xylene of PE or iPP was poured and uniformly spread on a preheated glass slide at around 130 °C. After the evaporation of xylene, a thin molten layer of the related polymer was then picked up by the motor‐driven cylinder with a drawing speed of 4–20 cm s^−1^ and highly oriented ultrathin polymer films of 30–60 nm in thickness and square centimeters in area were collected by glass slides. Samples used for studying the epitaxial crystallization of the homopolymers, copolymers, and blends were prepared by spin‐coating the 0.5 wt.% chloroform solution of them onto surface of the highly oriented PE or iPP substrate films. The double‐layered thin films were used either directly after spin‐coating or heat‐treated at 80 °C for 10 min to erase their previous thermal history and then cooled down to desired temperature for a complete isothermal crystallization.

### 1H NMR Characterization

^1^H NMR spectra were recorded on a Bruker AV400 (400 MHz) spectrometer. Chemical shifts (*δ*) were given in parts per million (ppm) relative to tetramethylsilane (TMS; *δ* = 0) as the internal reference. ^1^H NMR spectra data were reported as chemical shift, relative integral, multiplicity (s = singlet, d = doublet, m = multiplet), coupling constant (J in Hz), and assignment.

### AFM Characterization

AFM images were collected on the Fastscan A instrument (Bruker) and analyzed by the Nanoscope software. For in situ study during heating process, a heating rate of 1 °C min^−1^ from room temperature to the desired temperature was used, and each AFM image was obtained after the sample had been held ≈7 min at the corresponding temperature. The scanning density was 512 lines per frame.

### TEM Characterization

For transmission electron microscopy (TEM) study, a JEOL JEM‐2100 with an accelerating voltage of 200 kV was used in this study. To minimize radiation damage by the electron beam, focusing was carried out on an area; then the specimen film was translated to an adjacent undamaged area for recording the images immediately.

### XRD Characterization

The 2D X‐ray diffraction (XRD) data were obtained on the beamline 1W2A of Beijing Synchrotron Radiation Facility (BSRF), Beijing, China. The wavelength of X‐ray was 0.154 nm. The measurement of 2D‐small angle X‐ray scattering (SAXS) data was performed at a Xenocs Xeuss 2.0 instrument equipped with a Linkmam THMS600 hot stage in a transmissive mode. The X‐ray exposure time was 100 s for every measurement and the heating rate of 1°C min^−1^.

### FTIR Characterization

The FTIR analysis was carried out by a Spectrum 100 FTIR spectrometer (PerkinElmer). Polarized FTIR was used to calculate the orientation function of oriented films. FTIR spectra in the wavenumber range from 3000 to 800 cm^−1^ were obtained by averaging 32 scans at 4 cm^−1^ resolution.

## Conflict of Interest

The authors declare no conflict of interest.

## Supporting information

Supporting Information

## Data Availability

The data that support the findings of this study are available from the corresponding author upon reasonable request.
