# Supplementary material for: Epitaxy‐Directed Self‐Assembly of Copolymers and Polymer Blends
Source: Adv Sci (Weinh). 2023 Nov 23;11(2):2207707. doi: 10.1002/advs.202207707 (PMC10787078; doi:10.1002/advs.202207707)
Supplement: Supplementary file 1 — Supporting Information [file ADVS-11-2207707-s001.pdf]

ADVANCED SCIENCE
Open Access

Supporting Information

for *Adv. Sci.*, DOI 10.1002/advs.202207707

Epitaxy-Directed Self-Assembly of Copolymers and Polymer Blends

Chunyue Hou, Junjie Wang, Peng Wang, Jiahui Cui, Shaojuan Wang, Rui Xin, Huihui Li, Xiaoli Sun, Zhongjie Ren* and Shouke Yan**

Electronic Supplementary Information

For

Epitaxy-Directed Autonomous Self-Assembly of Copolymers and Polymer Blends

Chunyue Hou,[1†] Junjie Wang,[2†] Peng Wang,[2] Jiahui Cui,[2] Rui Xin,[1] Jian Hu,[1] Huihui Li,[2] Xiaoli Sun,[2] Zhongjie Ren,[2]* Shouke Yan[1,2]**

Dr. C. Hou[1], Dr. J. Wang[2], Mr. P. Wang[2], Mrs. J. Cui[2], Dr. R. Xin[1], Dr. J. Hu[2], Dr. H. Li[2], Prof. Dr. X. Sun[2], Prof. Dr. Z. Ren[2], Prof. Dr. S. Yan[1,2]

[1] Key Laboratory of Rubber-Plastics, Qingdao University of Science & Technology, Qingdao 266042, China.

[2] State Key Laboratory of Chemical Resource Engineering, Beijing University of Chemical Technology, Beijing 100029, China

E-mail: S. Yan, skyan@qust.edu.cn or skyan@mail.buct.edu.cn

X. Sun, xiaolisun@mail.buct.edu.cn

Z. Ren, renz@mail.buct.edu.cn

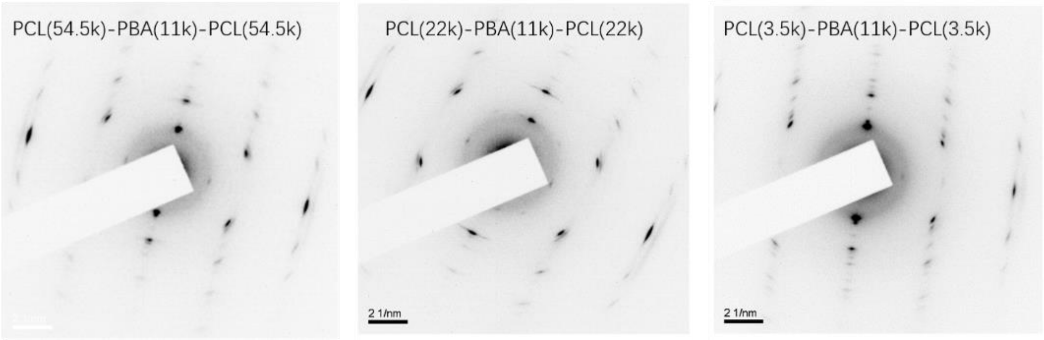

Figure S1. The electron diffraction patterns of PCL-*b*-PBA-*b*-PCL with different PCL block lengths as indicated in each image. The appearance of diffraction spots of all components demonstrates that the epitaxy-directed autonomous self-assembly of PCL-*b*-PBA-*b*-PCL block copolymer on oriented PE substrate is block length independent.

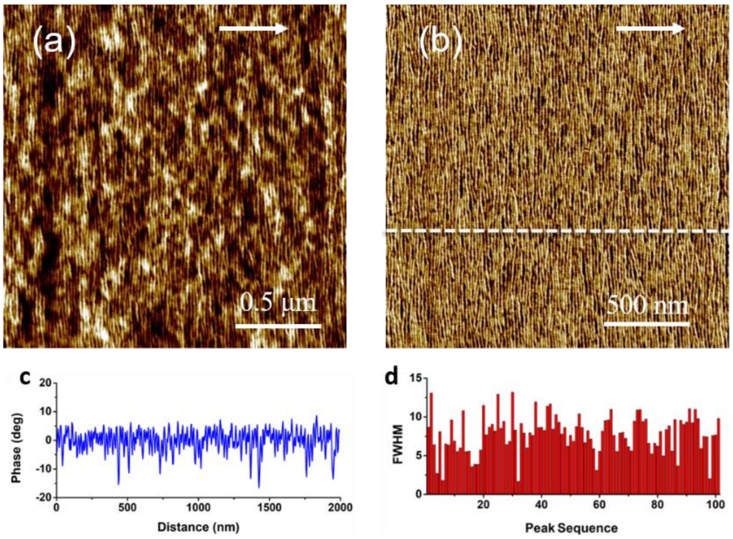

Figure S2. AFM height (a) and phase (b) images of PCL(13.5k)-b-PBA(11k)-b-PCL(13.5k) triblock copolymer grown isothermally on oriented PE substrate at 32.5 °C. (c) The height profile corresponding to the white line shown in part (b). (d) The FWHM distribution obtained from part (c). The molecular chain direction of the highly oriented PE substrate film is indicated by the white arrows.

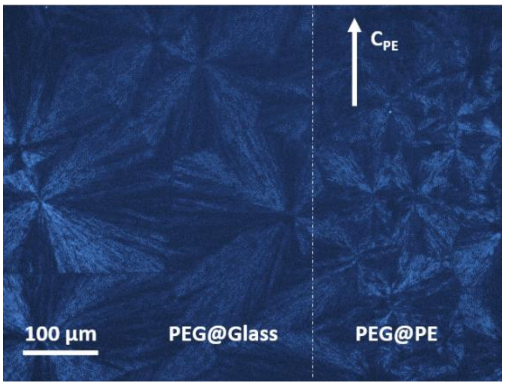

Figure S3. An optical micrograph of a PEG homopolymer isothermally grown at 40 °C on glass side partially covered by an oriented PE film taken under crossed polarizers. The PE film is located in the right part of the image. The boundary line and molecular chain direction of PE film are indicated by a dashed white line and a white arrow, respectively. Even though the reduced PEG spherulite size on PE substrate reveals the tiny influence of oriented PE film on the crystallization of PEG, the same spherulitic morphologies of it on both glass slide (left) and PE (right) surfaces demonstrate the lack of epitaxial ability of PEG on PE substrate.

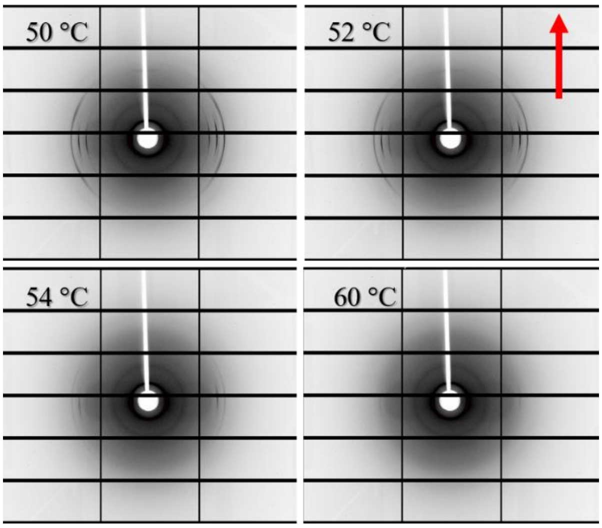

Figure S4. WAXD patterns of PCL(50k)-*b*-PEG(1.9k) diblock copolymer grown epitaxially on highly oriented PE substrate at 40 °C after melting at 80 °C for 10 min, which were taken in the heating process at different temperature as indicated. The arrow shows the molecular chain direction of oriented PE film.

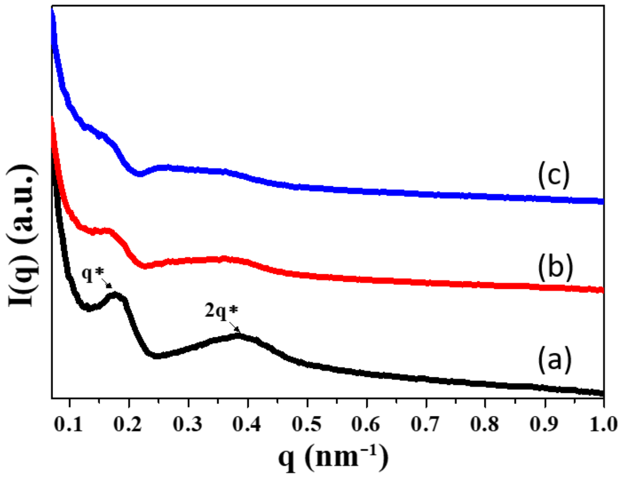

Figure S5. 1D-SAXS profiles obtained from the 2D-SAXS patterns shown in Figure 4.

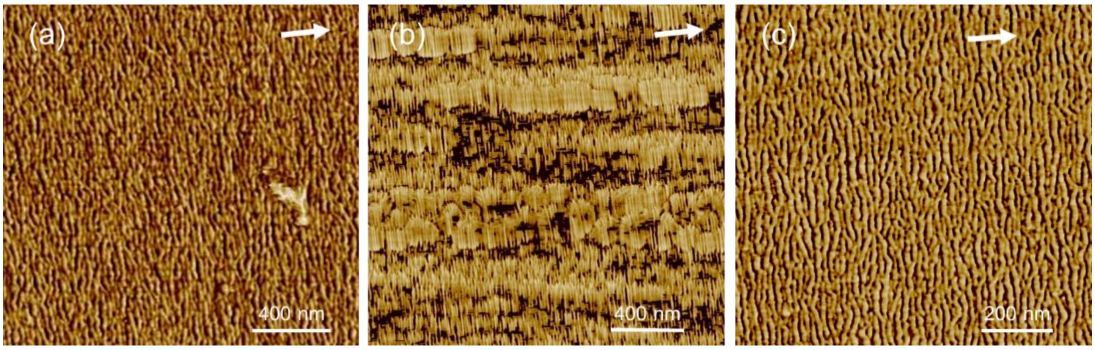

Figure S6. AFM phase images of (a) PEG(1.9k)-PCL(10k), (b) PEG(1.9k)-PCL(20k), and (c) PEG(20k)-PCL(20k) copolymers crystallized on oriented PE substrates isothermally at 40 °C

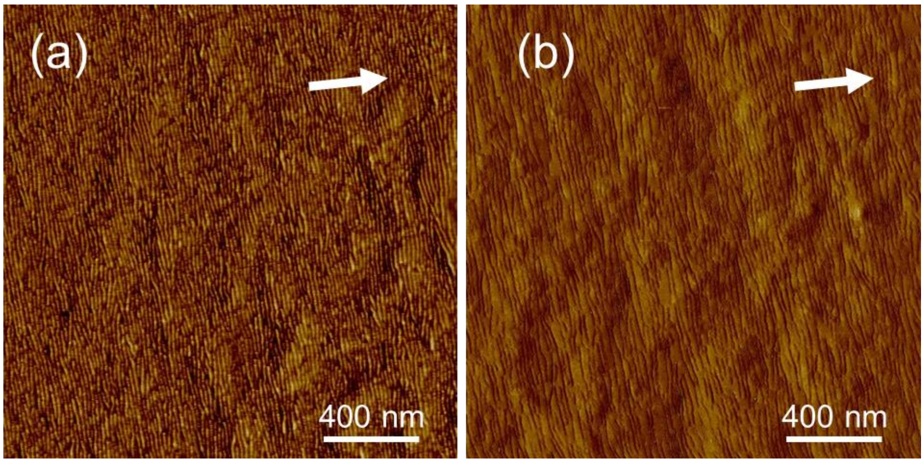

Figure S7. AFM phase images of PCL/PBA 50/50 blends crystallized on oriented PE substrate at (a) 30 °C and (b) 40 °C, respectively. The white arrows indicate the PE chain direction.

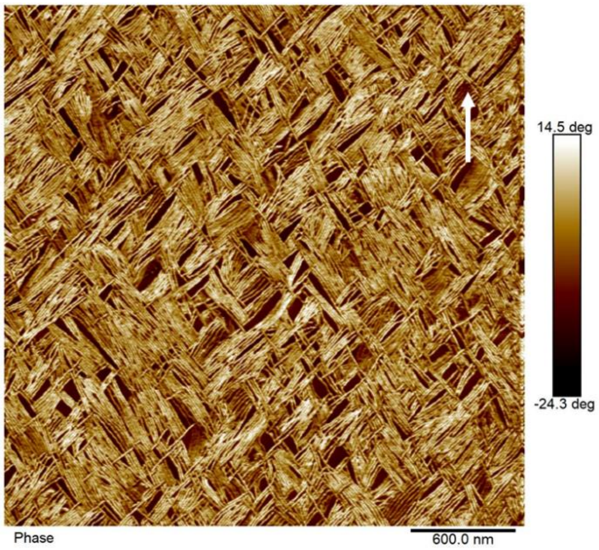

Figure S8. AFM phase image of PCL epitaxially crystallized on oriented iPP substrate from solution. The arrow shows the molecular chain direction of oriented iPP substrate.

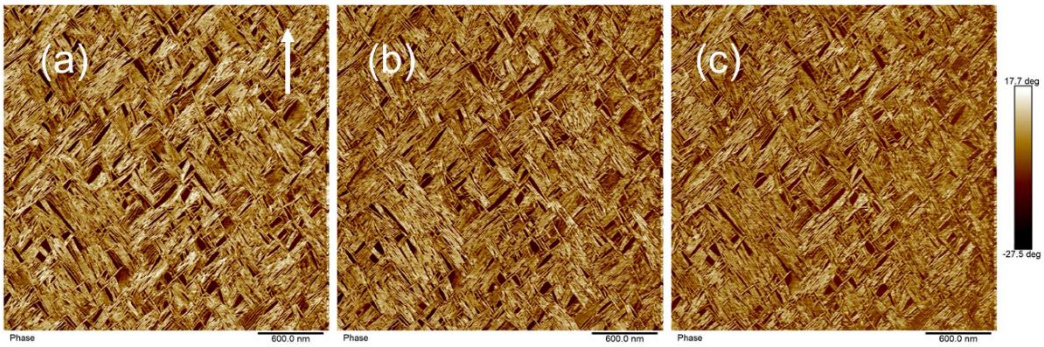

Figure S9. AFM phase image of PCL epitaxially crystallized on oriented iPP substrate from solution shown in Figure S7 after annealing at (a) 50 °C, (b) 52 °C, and (c) 54 °C.

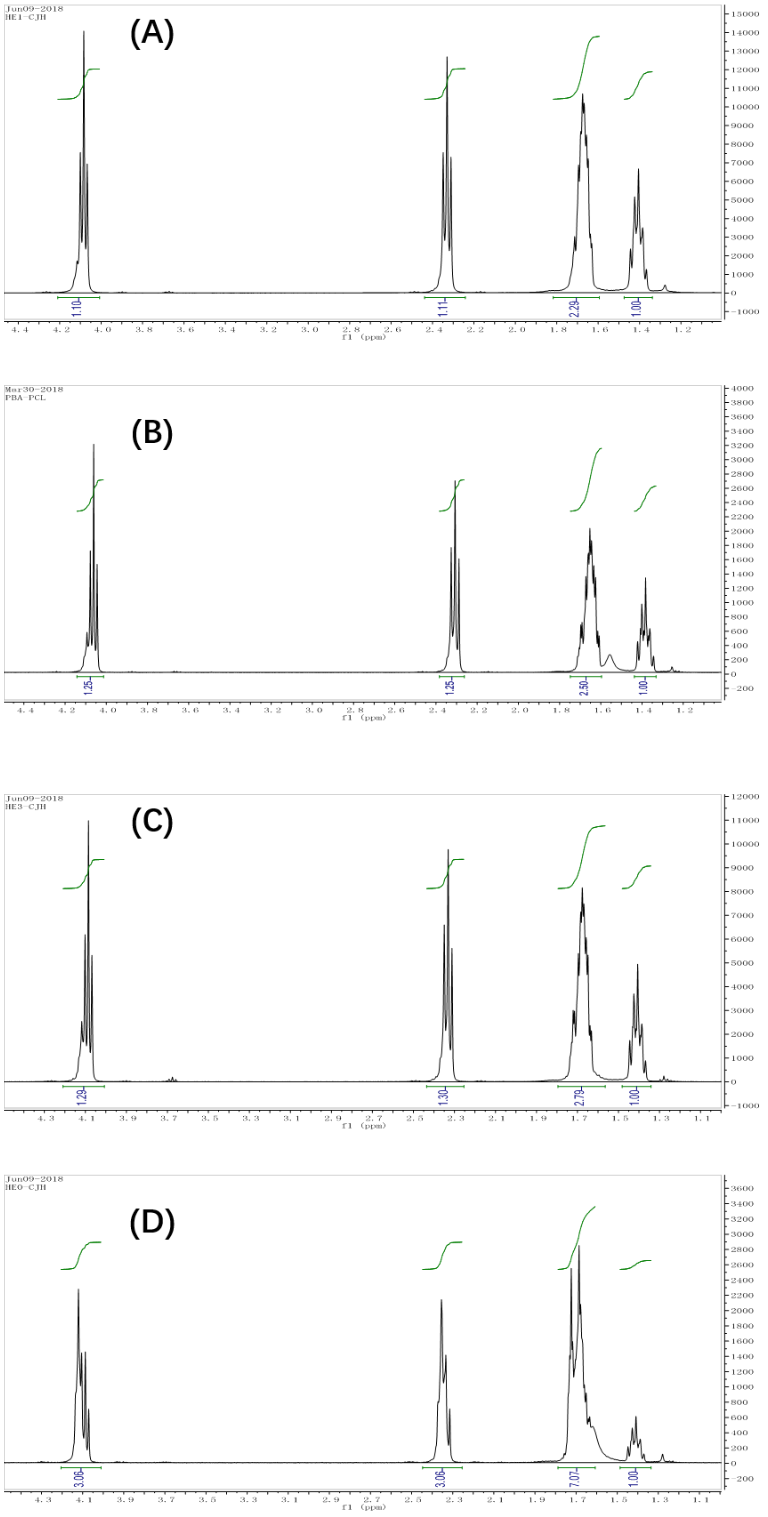

Figure S10. ^{1}H NMR of (A) PCL(54.5k)-*b*-PBA(11k)-*b*-PCL(54.5k), (B) PCL(22k)-*b*-PBA(11k)-*b*-PCL(22k), (C) PCL(13.5k)-*b*-PBA(11k)-*b*-PCL(13.5k), and (D) PCL(3.5k)-*b*-PBA(11k)-*b*-PCL(3.5k).

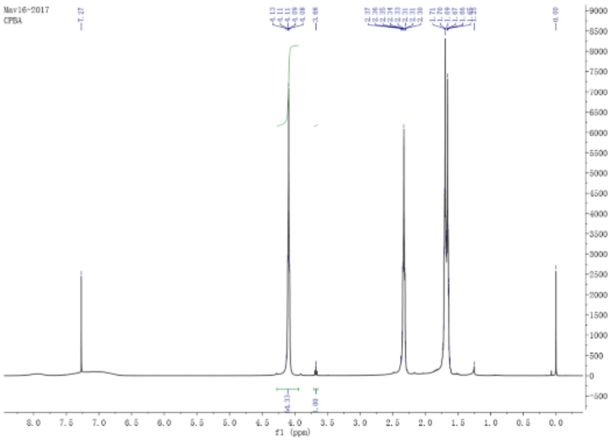

Figure S11. ^{1}H NMR of poly(butylene adipate) diol.

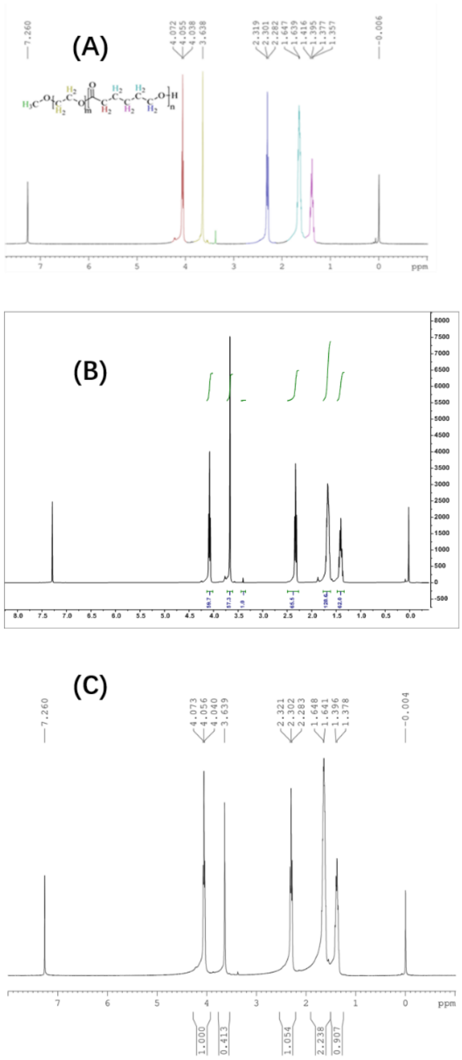

Figure S12. ^{1}H NMR of (A) PEG(1.9k)-*b*-PCL(5k), (B) PEG(1.9k)-*b*-PCL(10k), and (C) PEG(1.9k)-*b*-PCL(20k).
